# Retrieval from long-term memory does not bypass working memory

**DOI:** 10.3758/s13414-025-03145-z

**Published:** 2025-08-22

**Authors:** Michael K. P. Mugno, Timothy J. Vickery

**Affiliations:** Department of Psychological and Brain Sciences, University of Delaware, Newark, DE USA

**Keywords:** Long-term memory, Working memory, Visual cognition, Long-term memory retrieval

## Abstract

Information retrieved from long-term memory (LTM) enters working memory (WM), and the amount of information that can be retrieved is constrained to the limits of WM (about three to four items; Fukuda & Woodman, *Proceedings of the National Academy of Sciences*, *114* (20), 5306-5311, 2017). Can LTM retrieval occur when WM is near capacity, without consequence to either the retrieved or the maintained information? Liu, Li, Theeuwes, and Wang (*NeuroImage*, *261*: 119513, 2022) presented evidence that even when WM is near capacity, LTM items could still be reported. They argue that retrieved LTM items can bypass WM. We investigated this further by introducing continuous reporting of retrieved information and WM contents to their paradigm. If retrieval bypasses WM, then there should be no impairment of report accuracy to either WM contents or LTM-retrieved information. In the first experiment, WM reports suffered when an LTM item was retrieved. In the second, we found that when WM was near capacity (four items), the fidelity of LTM reports suffered compared to when WM was not (two items or no items). Additionally, WM contents were reported with lower fidelity when an LTM item was retrieved compared to a WM-only condition, under both two-item and four-item WM load. We conclude that LTM retrieval does not bypass WM.

## Introduction

Human memory is often categorized into unique but tightly interconnected components. As far back as James (1890), it has been theorized that memory consists of a short-term component, where information is briefly held online to be acted upon, and a long-term component, where information is stored for later use. Atkinson and Shiffrin’s (1968) model built on this by establishing mechanisms for the storage and retrieval of memories, and these act as pathways between the two components. To store information into long-term memory (LTM), it must first enter working memory before being encoded (Baddeley & Hitch, 1974), and to retrieve information from LTM, it must first enter WM, to then be used (Atkinson & Shiffrin, 1968; James, 1890).

This was supported by Fukuda and Woodman (2017), who provided evidence that items retrieved from LTM are constrained by the limits of WM. In their task, they first had subjects train to associate arrays of color items (set sizes 1, 2, 4, and 8 items) with letter cues. Next, they cued retrieval by presenting one of those letters and asked subjects to report one of the colors from the associated array. They measured subjects’ alpha band oscillations using electroencephalography (EEG) to track patterns of electrophysical activity. Alpha oscillations are known to index WM load (Hu et al., 2019; Jenson et al., 2002), making them a useful marker for determining whether LTM items enter WM. (However, they are also associated with other related constructs; see Klimesch, 2012, for a review.) If LTM items do enter WM, alpha patterns should resemble those seen when WM items enter WM. They found that alpha does increase with each added LTM item, and importantly, subjects’ memory for items reached a ceiling at four items, which follows the traditional capacity limit given for VWM (three to four items; Baddeley, 2003; Cowen, 2001; Vogel & Machizawa, 2004). This correspondence suggests that LTM items are indeed entering WM, as retrieval is constrained by the capacity of WM.

Fukuda and Woodman’s task was performed when WM was empty, but is retrieval still possible when WM is *full*? Liu et al. (2022) presented evidence that, yes, retrieval is still possible; they presented subjects with a simultaneous WM + LTM task like that of Fukuda and Woodman and found that more items could be remembered compared to a baseline WM-only condition, suggesting that the retrieved LTM items were present online along with the WM items. However, they claimed that the retrieved LTM items were not entering WM per se, but rather *bypassing* WM and being held online in a different state. They claim that because Cowan’s K increased in the WM + LTM condition beyond the WM-only baseline, assuming the limit to WM is four items, then the LTM item must not be entering WM.

This logic follows theories of WM that describe it as having discrete “slots” that items occupy, and there is a limit to the number of items that can be held (again, the traditional three to four items). Recent evidence has challenged this quantized view of WM, suggesting that WM is more flexible and capable of representing information in varied states of precision based upon task demands (Bays & Husain, 2008). These models of WM describe a limited amount of “resources” that can be flexibly allocated toward items held online (Alvarez & Cavanagh, 2004; Bays et al., 2009; Gorgoraptis et al., 2011; Wilken & Ma, 2004). As the amount of information increases in the system, fewer resources will be allocated toward new incoming information, and vice versa. These theories propose that the number of items that can be held online is not set at three to four items, but can increase beyond this limit; however, average item fidelity decreases dramatically as the system encodes additional information.

**Fig. 1:**
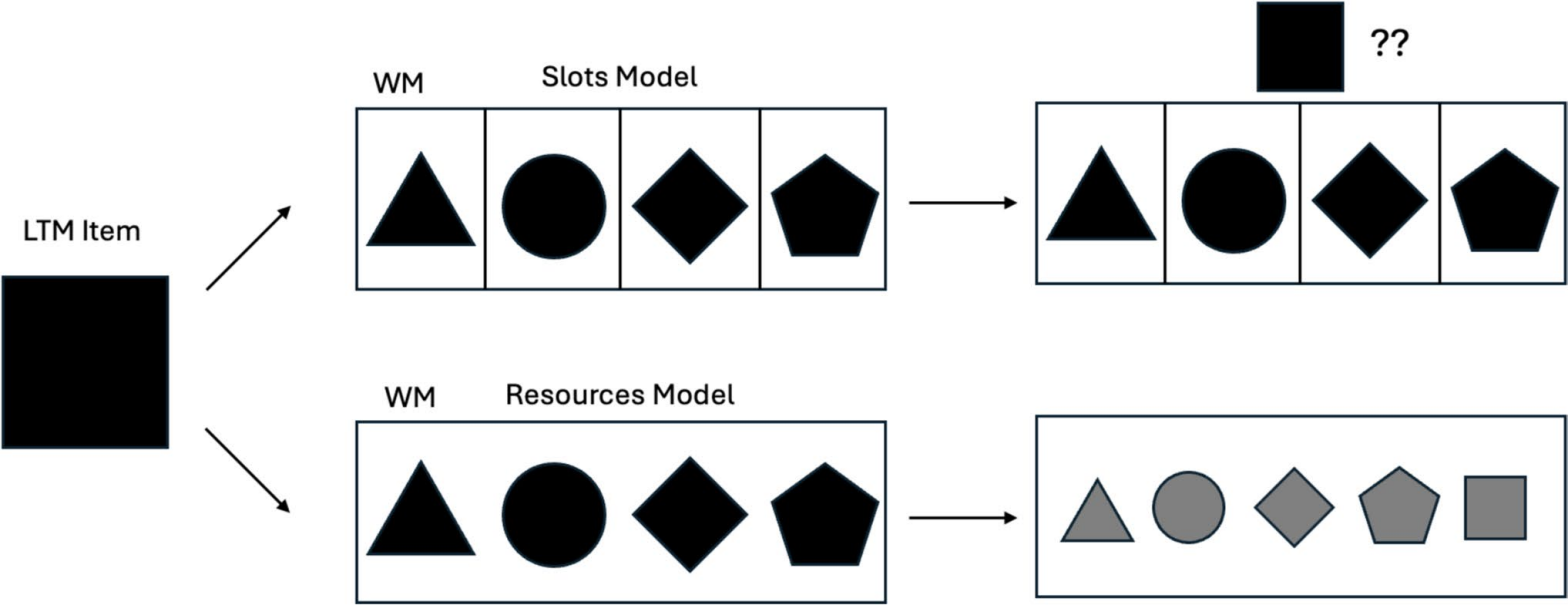
Comparing how long-term memory (LTM) items are retrieved according to the slots and resources models of working memory (WM). In the slots model, if the limited number of slots are filled, where would the retrieved LTM item be placed? In the resources model, the LTM item siphons resources from the already-present items in WM, leading to decreased fidelity of items

Therefore, despite apparent increases in K under WM+LTM versus WM-only, it remains possible that the LTM item indeed enters WM when it is full, albeit at the cost of the mean fidelities of some or all items. Whereas a slots model would leave the retrieved LTM item in a nebulous state or would replace a WM item in one slot, a resources model would accommodate the LTM item (Fig. 1). This would explain the finding that K increases when retrieval is cued.

Here we argue that when WM is near capacity, retrieved information from LTM is still encoded in WM, albeit at a cost to the fidelity of items. To improve the chances of observing such an effect, we used continuous reporting (in contrast to the forced-choice task used by Liu et al.) to robustly observe the interaction between retrieved LTM items and those already present in WM. Item fidelities can be interpreted from the accuracy with which a target item is reported, where subjects reported color items as closely to their true hue as they could on a color wheel. From there, the error in degrees can be measured from the target color’s angle on the color wheel and the reported color’s angle. Reports with smaller error would indicate that the item was represented with better fidelity within WM, while reports with more error would be interpreted as items having a weaker representation in WM.

If retrieved LTM items enter a WM that is already full, then we should see the fidelities of both WM and LTM items diminish compared to baseline when an LTM item is retrieved. This interaction would indicate that the LTM item is indeed entering WM, as it is siphoning resources from the already-present WM items and weakening their fidelities.

The two experiments here used a design similar to that of Liu et al. (2022). In both experiments, subjects were first given a training session to encode color-letter associations into LTM, followed by the main task. In Experiment 1, subjects were given a baseline WM-only condition where subjects were presented with two or four items to hold online, followed by a probe where subjects reported one of the items on a color wheel. Another condition combined both a WM task and an LTM task (WM + LTM), where subjects were cued for retrieval from LTM followed by the WM task, and both the LTM item and one of the WM items were probed. In Experiment 2, a baseline LTM-only condition was included to more accurately observe LTM fidelity changes compared to the WM + LTM condition, along with a reordering of stimulus presentation order to more accurately reflect the hypotheses (where WM items were presented first to fill WM before LTM retrieval was cued). To preview our results, we found that in Experiment 1, WM item fidelities did suffer following retrieval compared to baseline, along with an effect of set size. In Experiment 2, we found that both WM and LTM item fidelities suffered following retrieval; however, for LTM items this effect was only seen when WM held four items and not in set size 2.

## Experiment 1

This experiment modeled that of Liu et al.’s (2022) second experiment. Subjects were first given an LTM training task, where they were shown a unique color item that corresponded with one of six possible locations followed by a prompt to report the color and a letter cue to associate the color with. In the main task, subjects were given two trial types: a WM-only task, were they were given two or four color items and were asked to report one of them, and a WM + LTM task, where they were presented with an LTM cue they previously learned followed by a WM task and a report of the LTM item and one of the WM items. Our results showed that the fidelity of WM items was affected by retrieval from LTM; however, the fidelity of LTM items did not change across conditions.

### Methods

#### Participants

Twenty-three subjects were recruited through the University of Delaware subject pool. The target sample size was 20 subjects in order to achieve adequate power to detect a medium-to-large effect size in our primary analysis of interest (a main effect of LTM retrieval on WM performance: effect size of η_p_^2^ = 0.17, with a power of.80; effect of WM set size on LTM performance: effect size of *d*_*z*_ = 0.67 with a power of.80). An additional three participants were recruited due to overscheduling. All subjects reported normal or corrected-to-normal vision. Procedures were ruled exempt by the University of Delaware Institutional Review Board.

#### Apparatus and stimuli

Subjects were tested approximately 50 cm from an ASUS VG24BQE 3D Vision monitor (1,920 × 1,080 resolution, 144 Hz). The experiment script was written in MATLAB 2023b using Psychtoolbox 3.0.19. Stimuli were colored circles (3.25˚ in diameter) that could be one of 360 colors, corresponding with the degrees on a color wheel. The four LTM items and their associated letter cues are as follows: yellow (RGB: 255,251,0), A; green (3,255,0), B; blue (0,1,255), C; and purple (254,0,255), D. On a given trial, each one was uniquely associated with one of six locations on the screen. All WM items were chosen randomly from 360° on the color wheel but could not be within 20° of each other or the LTM item on a given trial (if one was presented).

#### Procedure

**Fig. 2:**
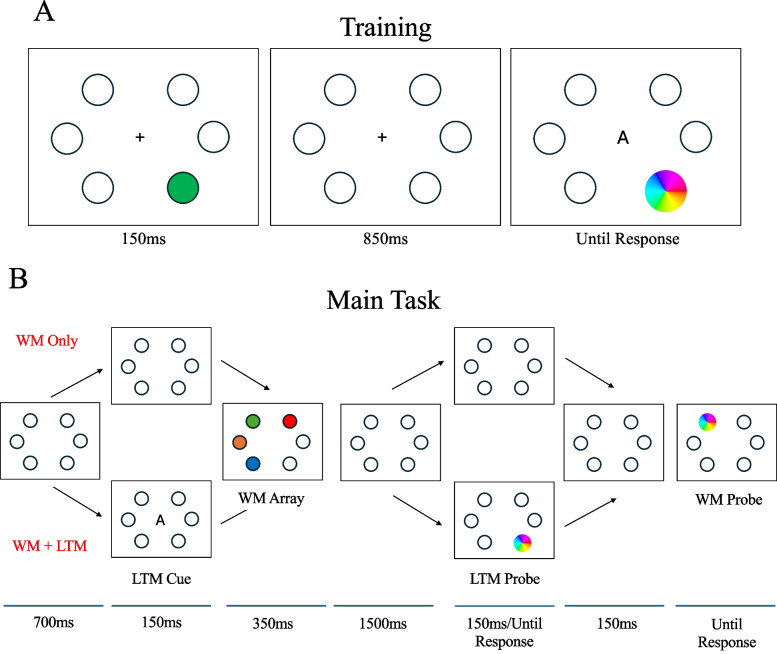
Procedure for Experiment 1. **A**) In the Training task, subjects are presented a color in one of the six set locations. Following a delay, a letter cue was presented in the center of the screen along with a color wheel presented around the location of the color. **B**) In the Main Task, there were two retrieval conditions (WM Only, WM + LTM) and two set sizes (two or four items). In the WM Only condition, subjects were presented with items to be held in memory. After a delay, a color wheel was presented around one of the locations where an item was displayed earlier. In the WM + LTM condition, subjects were given one of the letter cues from the training and asked to recall the color associated with that letter. They were then given a WM array, followed by probes to report the LTM colors and one of the WM colors

Training and test procedures are depicted in Fig. 2.

### Training

Six black outlined circles used as possible locations were placed 6˚ of visual angle away from the center of the screen, along with a fixation cross (0.3˚). After a brief delay (700 ms), one of the four LTM items was displayed at its associated location for 150 ms. Following another 850-ms delay, a color wheel was presented at the location followed by the associated letter presented above the fixation cross. Subjects were tasked with clicking the color on the wheel they matched their memory for that item. Finally, they were given feedback for 2,000 ms.

### Main task

The same six outlined locations from the Training Phase were used. The experiment used a 2 × 2 design with factors WM Set Size, with set sizes 2 and 4, and Retrieval, with a WM Only condition and a WM + LTM condition. Set Size was blocked, where all trials in a block had the same set size, and Retrieval conditions were randomized within a block. The order of blocks was counterbalanced between subjects. There were eight blocks (four of each type) with 32 trials in each block (16 trials of each Retrieval condition), for a total of 256 trials.

In the WM Only condition, the trial started with a fixation presented for 850 ms. The WM stimuli were presented for 500 ms in random locations, followed by another 3,500-ms delay. Finally, the color wheel surrounded one of the locations and subjects were asked to report the color presented in that location as accurately as possible.

In the WM + LTM condition, the trial started with a fixation presented for 700 ms, followed by a letter cue presented for 150 ms. Following a 350-ms delay, the WM items were displayed for 500 ms (note that none of the items were presented in the location of the LTM item). After another 1,500-ms delay, a color wheel was presented around the location of the LTM item, and after another 150-ms delay after subjects responded, a color wheel highlighted one of the WM item locations.

### Results

#### Training

Subjects’ mean degree error in reporting was 8.92°. This mean stayed stable over the course of the training task.

#### Main task

A 2 × 2 ANOVA on conditions set size (two items, four items) and retrieval (WM Only, WM + LTM) was conducted on mean degree error for reports of WM items. There was no significant interaction effect, *F*(1,22) < 1; however there were significant main effects of set size, *F*(1,22) = 121.70, *p* <.001, η_p_^2^ =.847, and retrieval, *F*(1,22) = 80.44, *p* <.001, η_p_^2^ =.785. Post hoc testing revealed that subjects’ reporting errors increased as set size increased, where errors increased by 21.10° as the number of items in WM arrays increased from two to four items, *t*(22) = 11.03, p <.001. Errors in WM item reports similarly increased by 9.77° when information from LTM was retrieved compared to when information was not cued for retrieval, *t*(22) = 8.97, *p* <.001.

**Fig. 3:**
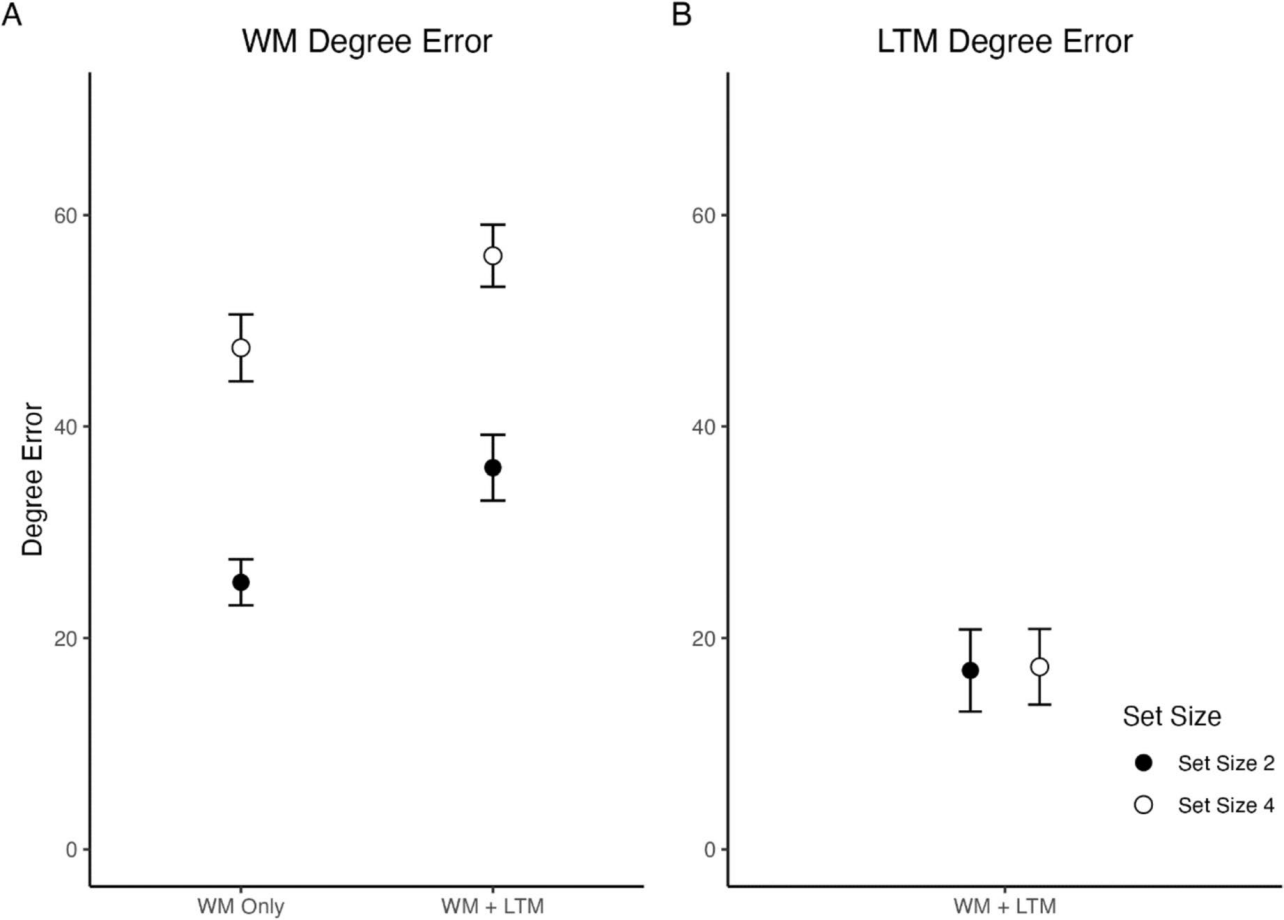
Experiment 1 results, working memory (WM) degree errors and long-term memory (LTM) degree errors. Subjects showed an increase in WM item report errors when LTM items were retrieved and also when set size increased from two to four items. LTM item reports did not significantly differ as set size increased. Error bars represent standard error of the mean (SEM)

**Fig. 4:**
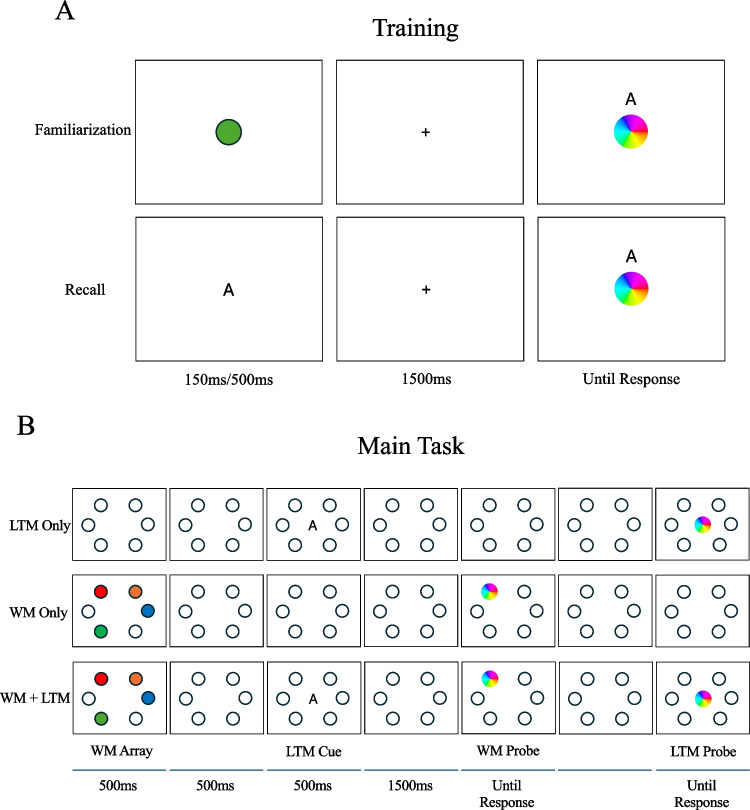
Procedure for Experiment 2. **A**) In the Familiarization block of the Training Task, subjects are presented a color in the screen center. Following a delay, a letter cue was presented along with a color wheel. In the Recall block, subjects were given one of the letter cues and asked to recall the color associated with it. After a delay, they were probed with a color wheel. **B**) In the Main Task, there were three retrieval conditions (LTM Only, WM Only, WM + LTM) and two set sizes (two or four items). In the LTM Only condition, subjects essentially repeated the recall task from training. In the WM Only condition, subjects were presented with items to be held in memory, and after a delay, a color wheel was presented around one of the locations where an item was displayed earlier. In the WM + LTM condition, subjects were first given a WM array followed by a letter cue. They were then probed with a WM item, and then the LTM item

**Fig. 5:**
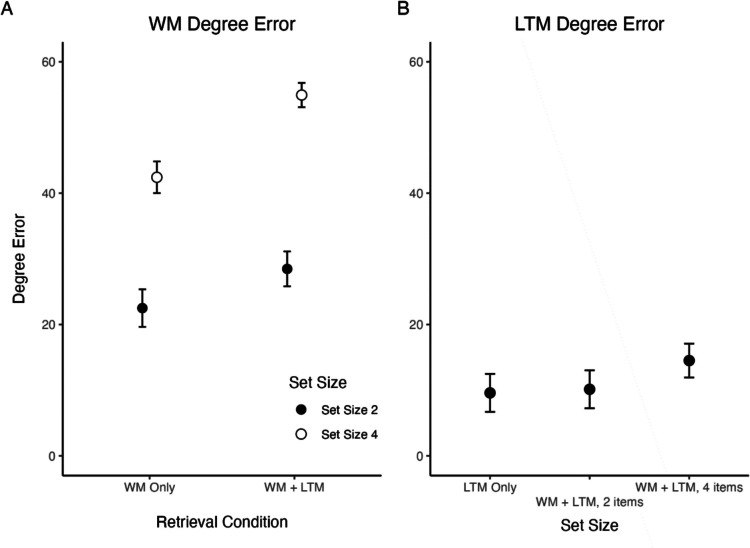
Experiment 2 results, Working memory (WM) degree errors and long-term memory (LTM) degree errors. Subjects showed an increase in WM item report errors when LTM items were retrieved and also when set size increased from two to four items. LTM item reports did not significantly differ between LTM Only and when WM held two items, but did when WM held four items. Error bars represent standard error of the mean (SEM)

Because there was no baseline condition to which the fidelity of retrieved LTM information could be compared (an issue that was addressed in the next experiment), a simple t-test was conducted between LTM item reports for set size conditions. There was no significant difference in retrieved LTM item reports between set sizes, *t*(22) = 0.25, *p* =.808 Figs. 3, 4 and 5.

## Discussion

In Experiment 1, subjects were first trained to associate colors with letter cues and locations in an array. In the main task, they were either given one of the letter cues to recall its paired color, or not given a retrieval cue. Following this, they completed a WM task in which they remembered either two or four items in the array. Finally, they either reported both the LTM item and one of the WM items, or only one of the WM items (when LTM was not cued).

The results revealed that the fidelity of items held in WM were affected by the introduction of retrieved LTM information. The WM item reports showed an effect of both the retrieval of an LTM item and an increase in set size, where the fidelities of WM items were weakened by the presence of a retrieved item along with an increase in set size.

As for the LTM item reports, the lack of a difference in errors between set sizes would indicate that LTM items are unaffected by how much information is held in WM prior to retrieval. However, this experiment does not feature a baseline condition for LTM item reporting analogous to the WM Only condition. It is difficult, and inappropriate, to interpret how LTM items are being reported without a proper baseline. This was addressed in the next experiment.

Another important limitation in this experiment was the order of stimulus presentation inherited by Liu et al., where LTM retrieval was cued before new WM items were presented. The research question here is whether LTM enters or bypasses WM *when it is near-capacity*, and thus the order of presentation of LTM cues followed by WM items would seem impractical in addressing this. Regardless of whether one follows the slots or resources models, the LTM item clearly occupies space in WM prior to it receiving more input from new stimuli (Fukuda & Woodman, 2017, show that retrieved LTM items do indeed enter WM when it is empty). In the following experiment, we addressed this by simply presenting subjects with WM items first before cueing LTM retrieval, and thus properly examining our hypotheses.

A final, subtle flaw in the implementation of Experiment 1 was that the color wheels were not randomly rotated on each trial. This may have had an impact on how subjects encoded the color-letter associations in the training phase. Instead of creating a color-letter-location association, it is possible that they created a *color wheel location*-letter-location association. In other words, subjects could memorize where to click on the color wheel when the letter cue was presented rather than the actual color itself. This might support less interference between LTM and WM items, as well.

Even with these limitations, Experiment 1 provided some evidence that LTM information does not bypass WM when it is near capacity, as reports of the WM items decreased in fidelity when LTM information was retrieved. Experiment 2 addresses the above-noted limitations.

## Experiment 2

The second experiment included significant changes to the design of the first experiment in order to address its limitations. Specifically, the presentation order is inverted so that WM items are presented first followed by cued LTM retrieval in order to more closely follow the hypotheses; it also includes an LTM Only trial condition to serve as a baseline for measures of LTM item fidelity, as well as random rotations of the color wheels in order to prevent associations between the location of reports on the color wheel and the letter cue. A final modification was to further simplify LTM retrieval by removing color-letter associations with locations on the stimulus array; instead, they were trained, cued, and reported in the center of the screen. Our results showed that LTM items did not lose fidelity compared to baseline when WM held two items, but significantly lost fidelity when WM held four items. Similarly, WM items lost fidelity when two items were held compared to baseline and weakened further when four items were held.

### Methods

Twenty subjects were recruited, consistent with Experiment 1. This was done to ensure sufficient power (80%) to detect a medium to large effect size (η_p_^2^ = 0.17). This effect size was far exceeded in the WM results of Experiment 1. The design was similar to Experiment 1 with the following modifications. First, color wheels were rotated on each trial. Additionally, the order of stimulus presentation was changed, with WM items being presented before LTM cueing. Finally, an LTM Only condition was included.

#### Procedure

##### Training

The first 25 blocks were used to familiarize subjects with the LTM items, and in the next 25 blocks subjects were asked to recall the items in order to properly encode the associations between the colors and cues. In the first half, after a 1,000-ms fixation delay, the LTM item was presented for 150 ms in the center of the screen, followed by another 1,500-ms delay. At report, the letter cue was displayed above the color wheel at screen center. In the second half, subjects were not given the LTM item first, but rather practiced recall by being given a letter cue and reporting its associated color. Starting with a 1,000-ms fixation delay, the letter cue was presented for 500 ms, followed by a 1,500-ms delay. A color wheel was presented at screen center, and after responding, subjects were given feedback for 2,000 ms.

##### Main task

There were again eight blocks (four of each Set Size), but with 36 trials in each block (12 trials per Retrieval condition) for a total of 288 trials. Just like the Training Phase, LTM cues and color wheels were presented at the center of the screen, further preventing subjects from associating location information rather than color information.

##### **LTM Only**

Trials started with a 1,500-ms delay, followed by a letter cue presented at center for 500 ms. After another 1,500-ms delay, the color wheel was presented at center.

##### **WM Only**

Trials started with a 500-ms delay, followed by the presentation of WM items for 500-ms. After another 500-ms delay, the color wheel was presented around one of the item locations.

##### **WM + LTM**

Trials started with a 500-ms delay, followed by the presentation of the WM items for 500 ms. After another 500-ms delay, the letter cue was presented for 500 ms, followed by a 1,500-ms delay. A color wheel was presented at center to report the LTM item, and after response, a color wheel was presented around one of the WM item locations.

### Results

#### Training

During the second part of training, in which subjects practiced associations with a recall task, accuracy did not significantly change from the first to the second half of training, *F*(1,18) = 0.85, *p* =.37). Subjects’ learning of the color-letter associations was rapid and held stable across the task, indicating strong consolidation of those memories. The mean error in reporting was 11.52°.

#### Main task

There are two dependent variables in this experiment, WM error and LTM error. Each was analyzed separately, as WM had 2 × 2 conditions and LTM had 1 × 3. LTM had one level of retrieval since LTM was cued for retrieval in both LTM Only and WM + LTM conditions – the LTM Only condition was essentially a 0-item set size. For WM error, a 2 × 2 ANOVA was conducted, while for LTM a one-way ANOVA was run.

The ANOVA on WM performance yielded a significant interaction effect between set size and retrieval, *F*(1,19) = 8.44, *p* =.009, η_p_^2^ =.319. It also found main effects for both set size, *F*(1,19) = 115.44, *p* <.001, η_p_^2^ =.831, and retrieval (WM Only, WM + LTM), *F*(1,19) = 66.72, *p* <.001, η_p_^2^ =.575. Further investigation with pairwise comparisons showed that reported WM degree error increased as set size increased by a mean difference of 15.42°, *t*(19) = 9.89, *p* <.001, and reported WM degree error also increased by 9.60° in the WM + LTM condition compared to the WM Only condition, *t*(19) =5.30, *p* <.001. Within a set size of two items, between the WM only and WM + LTM conditions, degree error increased by 5,85°, *t*(19) = 2.99, *p* =.007, and similarly within a set size of four items, degree error increased by 13.34°, *t*(19) = 5.70, *p* <.001*.* Within the WM Only condition, WM degree error increased by 19.38° as set size increased, *t*(19) = 7.54, *p* <.001. Finally, within the WM + LTM condition, as set size increased from two to four items, degree error increased by 26.87°, *t*(19) = 10.11, *p* <.001.

The one-way ANOVA on LTM was significant, *F*(1,19) = 7.48, *p* = 0.010 η_p_^2^ =.293 (corrected for sphericity using the Greenhouse-Geisser method). Further investigation with pairwise comparisons showed that reported LTM degree error was not significantly different between LTM Only and when WM held two items, *t*(19) = 0.21, *p* = 0.836. When WM held four items, however, there was a significant increase in errors by 4.73° compared to LTM Only, *t*(19) = 2.32, *p* = 0.026. Finally, there was a significant increase in errors by 4.26° between when WM held two items and four items, *t*(19) = 1.72, *p* = 0.03.

### Discussion

The purpose of Experiment 2 was to address limitations of Experiment 1, including the order of stimulus presentation, a lack of a baseline to compare reports of LTM to, and issues with the training phase of the task.

These results lend strong evidence that retrieved LTM information is entering WM even when it is near capacity. The errors in reporting LTM items were significantly greater compared to baseline when WM held four items, and comparatively, the errors of WM item reports were dramatically greater than baseline. Therefore, both LTM and WM fidelities suffered when WM was near-capacity and information was retrieved from LTM. We also observed that when WM held two items, WM item report errors were significantly greater than baseline, but this was not observed in LTM item reports, where its slight increase in errors was not significant. Why are the fidelities of LTM items left relatively intact while the fidelities of WM items suffer? There could be a few explanations for this. It is possible that simply having the LTM task nested within the WM task degraded the fidelity of those WM items. A further investigation of this would simply repeat this experiment with a condition in which the WM task was nested within the LTM task. This is essentially the task in Experiment 1, but because that experiment did not include a baseline, an analysis of this nesting effect would not be possible. This decrease in WM fidelity at two items might also come from retrieved information being prioritized within WM, which would also see a siphoning of more resources towards the LTM information since it is seen as more important than the other items.

### General discussion

The purpose of this study was to investigate whether information retrieved from LTM enters WM when it is near capacity. It was proposed that if LTM items are encoded into WM, then both WM and LTM item fidelities would suffer as resources are siphoned from items already being stored in WM to accommodate a new LTM item. In the first experiment, WM item fidelities suffered when LTM items were cued for retrieval, especially when the number of WM items increased. This would indicate that WM items were being affected by this retrieval; however, without a control condition, it was unknown whether LTM items lost fidelity due to sharing resources with WM items. When this was corrected in the second experiment, both WM and LTM items lost fidelity. Interestingly, LTM items only lost fidelity when WM was filled with four items, but did not significantly lose fidelity when WM had two items online. From these results, we concluded that LTM items are indeed entering WM during recall, reducing available resources from the items currently being held online when retrieval occurs.

Our findings align with Liu et al. (2022) where they demonstrate that retrieval from LTM remains possible even when WM capacity is exceeded. However, we do not find evidence for their claim that LTM items bypass WM. They argue that it is not possible for LTM items to enter WM in the four-item condition because WM is filled at four items. We demonstrate the contrary, with our results implying that the capacity limit of WM is not on the number of items that are held online, but rather the amount of resources that can be allocated to each item (Bays & Husain, 2008; Oberauer & Eichenberger, 2013).

Our results are far more consistent with long-standing models of LTM than the findings of Liu et al. (2022). WM and LTM have long been viewed as deeply related even when conceptualized as separated stores, with LTM dependent upon WM for both encoding and retrieval (Atkinson & Shiffrin, 1968; Baddeley & Hitch, 1974; James, 1890). More recently, “embedded-component” models by Cowan (1999, 2001), Oberauer (2002, 2009), and others frame WM as deeply intertwined with LTM. For example, Oberauer’s conceptualization, building upon Cowan’s theory, involves activated regions of long-term memory, a resource-limited “region of direct access” that is essential for both access to WM contents and retrieved LTM contents, and limited focal attention that operates on items in the region of direct access. In these accounts, retrieved representations and those currently held in VWM would be forced to interact, compete, and likely interfere with one another within the region of direct access and due to shared limitations based on attention.

Our results are also consistent with other recent empirical work (beyond Fukuda & Woodman, 2017, as discussed in the *Introduction*), which supports the existence of interactions between VWM, attention, and LTM. VWM and attention are key to understanding effects on LTM representations that occur at the time of encoding or retrieval (Maxcey et al., 2015; Sundby et al., 2019). For example, comparisons occurring between perceived items and those stored in VWM can distort VWM representations (Fukuda et al., 2022) and those distortions persistently affect representations in LTM, as well (Saito et al., 2023). Furthermore, evidence suggests that LTM resources are recruited by WM to enhance and supplement WM processing (Adam et al., 2024; Mathyet al., 2024). Our findings that items retrieved from LTM interact robustly with items held in VWM contribute to the growing evidence that VWM and LTM are deeply intertwined.

A surprising observation in Experiment 2 was that the fidelities of WM items were significantly impacted by retrieval regardless of set size (two or four items), while the fidelity of LTM items was only impacted when set size was four items. Could information retrieved from LTM items be given precedence over what is being held in WM? Prior research demonstrates that the most task-relevant stimuli are given precedence, and therefore more resources allocated to their representations (Gorgoraptis et al., 2011; Maxcey-Richard & Hollingworth, 2013). It is possible that the task design, in which LTM retrieval was cued after WM was filled, may have inadvertently produced this effect and given the highest relevance to the LTM item. One could also argue that the results of Experiment 1 lend support to this, since the LTM item was cued before WM items were displayed and the results showed that errors in reporting LTM items did not differ between set sizes two and four. In this case, the LTM item is the first thing to fill WM (Fukuda & Woodman, 2017), with precedence already given by its cueing, and therefore it will be afforded some sort of buffering against its resources being recruited in favor of the new WM items. This has not been tested with retrieved LTM items specifically. Studies of prioritization within WM itself, however, have shown that items can and will be prioritized, resulting in more robust representations (Myers et al., 2017, 2018). Further investigation should address whether retrieved LTM items are specifically prioritized in WM compared to more general WM items. Past work suggests that the fidelities of LTM and WM items are nearly identical, and therefore retrieved LTM items are not inherently more robust than WM items (Miner et al., 2020).

It is also possible that this phenomenon is caused simply by the demand to recall precise LTMs, especially when the LTM task is nested within the WM task in Experiment 2. If the order of probes were reversed, it is possible that LTM item fidelities may not be as robust. It should also be noted that WM item fidelities were especially weak; again, this is likely due to the difficulty of reporting a precise memory of one of up to four items in WM.

The retrieved LTM item in our experiments can be viewed as essentially acting as a third or fifth item being encoded into WM. When it acted as a third item (WM set size 2) the fidelity of the LTM item did not significantly weaken, which would not be surprising as three items are typically observed to be held in WM with little issue (Bays et al., 2008; Luck & Vogel, 1997). When acting as a fifth item, however, we observe the fidelity of the LTM item weakening significantly. Future investigation should include WM-only set sizes of three or five items to compare item fidelity to the two- and four-item WM + LTM conditions.

As discussed in the *Introduction*, we used continuous reports to more precisely measure item representations’ fidelities in WM. Mixture models and swap models, as proposed by Zhang and Luck (2008) and Bays et al. (2009), are often used to account for errors due to random guessing, interference between items in WM, and reporting a color that was encoded but for a different location than the one probed. In the current context, we were agnostic to the source of increased error, because regardless of whether LTM retrieval caused lower precision or higher guess rate, either effect would imply interference in WM processes. Indeed, some subjects might have strategically lowered precision, while others might increase guess rates (by dropping WM items), which would cloud the results of such modeling. For this reason, and because the low number of trials per condition in our design would challenge model-fitting, we decided to forego mixture modeling in the current paper. Future work on this topic should address these limitations.

As we employed a dual-task paradigm during retrieval, other results relating LTM retrieval under divided attention (DA) are relevant to our findings. Previous evidence suggests that items take longer to be retrieved under DA, but that their accuracies are minimally affected (Craik et al., 1996; Naveh-Benjamin et al., 1998). However, Dodson et al. (1998) found diminished accuracy for specific-source information (i.e., fine-grained information) rather than partial-source information (i.e., course information). In the context of this study, it is possible that because attention is divided at retrieval (in the WM + LTM conditions), report errors increase at higher set sizes as more attention is drawn away from specific information. That is, subjects can accurately report the color category of a retrieved item (e.g., blue or red) but struggle to report the specific hue of the item in that category (e.g., sky blue or maroon). This affects conclusions drawn based upon the fidelity of LTM items retrieved under heavy attentional load. Notably, however, we principally and reliably see effects of LTM retrieval upon WM performance, and that alone implies that LTM information enters WM even under heavy load. Further, our response tasks were unspeeded, lightening the burden of DA on LTM performance. It may be worth further investigation of the role of DA in the effects we observed, but it does not affect our principal conclusions, drawn from the impact of LTM retrieval upon WM performance.

We note briefly that, because of the circular nature of the response space, no more than two items could be 20° from the LTM item, and no WM item could have more than two competing items 20° from themselves. It is possible that due to the tight constraints of the response space, the representations of items that are held online may interfere with each other because of their relative distances being close together. Critically, however, such competition would presumably occur within WM. Thus, if worse error rates occur because of the increased competition due to the LTM retrieval, then this is also evidence that LTM items are entering WM, rather than bypassing WM.

## Conclusion

Overall, evidence from these two experiments shows that information retrieved from LTM still enters WM when it is near capacity, albeit to the detriment of the fidelity of all items. We argue that information retrieved from LTM *can* and *will* enter WM even when it is near capacity by reducing resources allocated to items already held in WM. This further supports theories that include a resource model of WM.

## Data Availability

All data and experiment scripts are available via the Open Science Framework at https://osf.io/q6me4/.

## Code availability

All analysis and experiment scripts are available via the Open Science Framework at https://osf.io/q6me4/.

## References

[CR1] Adam, K. C. S., Zhao, C., & Vogel, E. K. (2024). Behavioral signatures of the rapid recruitment of long-term memory to overcome working memory capacity limits. *Memory & Cognition,**52*(8), 1816–1832. 10.3758/s13421-024-01566-z 38744775 10.3758/s13421-024-01566-z PMC11915716

[CR2] Alvarez, G. A., & Cavanagh, P. (2004). The capacity of visual short-term memory is set both by visual information load and by number of objects. *Psychological Science,**15*(2), 106–111. 10.1111/j.0963-7214.2004.01502006 14738517 10.1111/j.0963-7214.2004.01502006.x

[CR3] Atkinson, R. C., & Shiffrin, R. M. (1968). Human memory: A proposed system and its control processes. *Psychology of Learning and Motivation, 2*, 89-195. Academic press.

[CR4] Baddeley, A. D., & Hitch, G. (1974). Working Memory (G. H. Bower, Ed.; Vol. 8, pp. 47–89). 10.1016/S0079-7421(08)60452-1

[CR5] Baddeley, A. (2003). Working memory: Looking back and looking forward. *Nature Reviews Neuroscience,**4*(10), 829–839. 10.1038/nrn1201 14523382 10.1038/nrn1201

[CR6] Bays, P. M., Catalao, R. F., & Husain, M. (2009). The precision of visual working memory is set by allocation of a shared resource. *Journal of Vision,**9*(10), 1511–1519. 10.1167/9.10.7 10.1167/9.10.7 PMC3118422 19810788

[CR7] Bays, P. M., & Husain, M. (2008). Dynamic shifts of limited working memory resources in human vision. *Science,**321*(5890), 851–854. 10.1126/science.1158023 18687968 10.1126/science.1158023 PMC2532743

[CR8] Cowan, N. (1999). An embedded-processes model of working memory. In A. Miyake & P. Shah (Eds.), *Models of working memory: Mechanisms of active maintenance and executive control* (pp. 62–101). Cambridge University Press.

[CR9] Cowan, N. (2001). The magical number 4 in short-term memory: A reconsideration of mental storage capacity. *Behavioral and Brain Sciences,**24*(1), 87–114. 10.1017/S0140525X01003922 11515286 10.1017/s0140525x01003922

[CR10] Craik, F. I. M., Naveh-Benjamin, M., Govoni, R., & Anderson, N. D. (1996). The effects of divided attention on encoding and retrieval processes in human memory. *Journal of Experimental Psychology: General,**125*(2), 159–180. 10.1037/0096-3445.125.2.159 8683192 10.1037//0096-3445.125.2.159

[CR11] Dodson, C. S., Holland, P. W., & Shimamura, A. P. (1998). On the recollection of specific- and partial-source information. *Journal of Experimental Psychology: Learning, Memory, and Cognition,**24*(5), 1121–1136. 10.1037/0278-7393.24.5.1121 9747526 10.1037//0278-7393.24.5.1121

[CR12] Fukuda, K., Periera, A., Saito, J., Tang, T., Tsubomi, H., & Bae, G.-Y. (2022). Working memory content is distorted by its use in perceptual comparisons. *Psychological Science,**31*(5), 816–829. 10.1177/09567976211055375 10.1177/09567976211055375 PMC13021042 35452332

[CR13] Fukuda, K., & Woodman, G. F. (2017). Visual working memory buffers information retrieved from visual long-term memory. *Proceedings of the National Academy of Sciences of the United States of America,**114*(20), 5306–5311. 28461479 10.1073/pnas.1617874114 PMC5441785

[CR14] Gorgoraptis, N., Catalao, R. F. G., Bays, P. M., & Husain, M. (2011). Dynamic updating of working memory resources for visual objects. *Journal of Neuroscience,**31*(23), 8502–8511. 10.1523/JNEUROSCI.0208-11.2011 21653854 10.1523/JNEUROSCI.0208-11.2011 PMC3124758

[CR15] Hu, Z., Barkley, C. M., Marino, S. E., Wang, C., Rajan, A., Bo, K., Samuel, I. B. H., & Ding, M. (2019). Working memory capacity is negatively associated with memory load modulation of alpha oscillations in retention of verbal working memory. *Journal of Cognitive Neuroscience,**31*(12), 1933–1945. 10.1162/jocn_a_01461 31418335 10.1162/jocn_a_01461 PMC8018693

[CR16] James, W. (1890). The principles of psychology. *Henry Holt and Co*. 10.1037/10538-000

[CR17] Jensen, O., Gelfand, J., Kounios, J., & Lisman, J. E. (2002). Oscillations in the alpha band (9–12 Hz) increase with memory load during retention in a short-term memory task. *Cerebral Cortex,**12*(8), 877–882. 10.1093/cercor/12.8.877 12122036 10.1093/cercor/12.8.877

[CR18] Klimesch, W. (2012). Alpha-band oscillations, attention, and controlled access to stored information. *Trends in Cognitive Sciences,**16*(12), 606–617. 10.1016/j.tics.2012.10.007 23141428 10.1016/j.tics.2012.10.007 PMC3507158

[CR19] Liu, B., Li, X., Theeuwes, J., & Wang, B. (2022). Long-term memory retrieval bypasses working memory. *NeuroImage,**261*, Article 119513. 10.1016/j.neuroimage.2022.119513 35882271 10.1016/j.neuroimage.2022.119513

[CR20] Luck, S. J., & Vogel, E. K. (1997). The capacity of visual working memory for features and conjunctions. *Nature,**390*, 279–281. 10.1038/36846 9384378 10.1038/36846

[CR21] Mathy, F., Friedman, O., & Gauvrit, N. (2024). Can compression take place in working memory without a central contribution of long-term memory? *Memory & Cognition,**52*(8), 1726–1736. 10.3758/s13421-023-01474-8 37882946 10.3758/s13421-023-01474-8

[CR22] Maxcey, A. M., Fukuda, K., Song, W. S., & Woodman, G. F. (2015). Using electrophysiology to demonstrate that cueing affects long-term memory storage over the short term. *Psychonomic Bulletin & Review,**22*(5), 1349–1357. 10.3758/s13423-015-0799-2 25604772 10.3758/s13423-015-0799-2 PMC4510034

[CR23] Maxcey-Richard, A. M., & Hollingworth, A. (2013). The strategic retention of task-relevant objects in visual working memory. *Journal of Experimental Psychology: Learning, Memory, and Cognition,**39*(3), 760–772. 10.1037/a0029496 22845068 10.1037/a0029496 PMC3855846

[CR24] Miner, A. E., Schurgin, M. W., & Brady, T. F. (2020). Is working memory inherently more “precise” than long-term memory? Extremely high fidelity visual long-term memories for frequently encountered objects. *Journal of Experimental Psychology: Human Perception and Performance,**46*(8), 813–830. 10.1037/xhp0000748 32324030 10.1037/xhp0000748

[CR25] Myers, N. E., Chekroud, S. R., Stokes, M. G., & Nobre, A. C. (2018). Benefits of flexible prioritization in working memory can arise without costs. *Journal of Experimental Psychology: Human Perception and Performance,**44*(3), 398–411. 10.1037/xhp0000449 28816476 10.1037/xhp0000449 PMC5868459

[CR26] Myers, N. E., Stokes, M. G., & Nobre, A. C. (2017). Prioritizing information during working memory: Beyond sustained internal attention. *Trends in Cognitive Sciences,**21*(6), 449–461. 10.1016/j.tics.2017.03.010 28454719 10.1016/j.tics.2017.03.010 PMC7220802

[CR27] Naveh-Benjamin, M., Craik, F. I. M., Guez, J., & Dori, H. (1998). Effects of divided attention on encoding and retrieval processes in human memory: Further support for an asymmetry. *Journal of Experimental Psychology: Learning, Memory, and Cognition,**24*(5), 1091–1104. 10.1037/0278-7393.24.5.1091 9747524 10.1037//0278-7393.24.5.1091

[CR28] Oberauer, K. (2002). Access to information in working memory: Exploring the focus of attention. *Journal of Experimental Psychology: Learning, Memory, and Cognition,**28*(3), 411–421. 10.1037/0278-7393.28.3.411 12018494

[CR29] Oberauer, K. (2009). Design for a Working Memory. In *Psychology of Learning and Motivation* (Vol. 51, pp. 45–100). Elsevier. 10.1016/S0079-7421(09)51002-X.

[CR30] Oberauer, K., & Eichenberger, S. (2013). Visual working memory declines when more features must be remembered for each object. *Memory & Cognition,**41*(8), 1212–1227. 10.3758/s13421-013-0333-6 23716004 10.3758/s13421-013-0333-6

[CR31] Saito, J. M., Duncan, K., & Fukuda, K. (2023). Comparing visual memories to similar visual inputs risks lasting memory distortion. *Journal of Experimental Psychology: General,**152*(8), 2318–2330. 10.1037/xge0001400 36951741 10.1037/xge0001400

[CR32] Sundby, C. S., Woodman, G. F., & Fukuda, K. (2019). Electrophysiological and behavioral evidence for attentional up-regulation, but not down-regulation, when encoding pictures into long-term memory. *Memory & Cognition,**47*(2), 351–364. 10.3758/s13421-018-0871-z 30341544 10.3758/s13421-018-0871-z PMC6401211

[CR33] Vogel, E. K., & Machizawa, M. G. (2004). Neural activity predicts individual differences in visual working memory capacity. *Nature,**428*, 748–751. 10.1038/nature02447 15085132 10.1038/nature02447

[CR34] Wilken, P., & Ma, W. J. (2004). A detection theory account of change detection. *Journal of Vision,**4*(12), Article 11. 10.1167/4.12.11 10.1167/4.12.11 15669916

[CR35] Zhang, W., & Luck, S. J. (2008). Discrete fixed-resolution representations in visual working memory. *Nature,**453*, 233–235. 10.1038/nature06860 18385672 10.1038/nature06860 PMC2588137

